# Knowledge of triage in the senior medical students in Shiraz University of Medical Sciences

**Published:** 2016-07

**Authors:** HOSSEIN MAHMOODIAN, RAZIE EGHTESADI, ATEFE GHAREGHANI, PARISA NABEIEI

**Affiliations:** 1 Department of Medical Ethics, Shiraz University of Medical Sciences, Shiraz, Iran;; 2 Shiraz University of Medical Sciences, Shiraz, Iran;; 3 Department of Emergency Medicine, Shiraz University of Medical Sciences, Shiraz, Iran;; 4 Education Development Center, Quality Improvement In Clinical Education Research Center, Shiraz University of Medical Sciences, Shiraz, Iran

**Keywords:** Emergency, Triage, Medical students

## Abstract

**Introduction:**

Triage is a response to the problem of overcrowding in Emergency Departments (EDs) and accuracy of decisions made by the triage unit affects the ultimate outcome of EDs. This study was conducted to evaluate the knowledge of triage among last year medical students in Shiraz University of Medical Sciences.

**Methods:**

This is a cross-sectional analytical study whose subjects were all the senior students of medicine (62) in the last year of medicine from January to June 2013 who attended emergency medicine course in the screen room of 2 University Hospitals. This questionnaire was designed in 3 sections including personal data, 15 questions on knowledge of triage and 10 case scenarios for triage decision making and completed by the students. Statistical analysis was performed in SPSS statistical software (version 14) using independent sample t-test, one way ANOVA, and Pearson correlation coefficient (p≤0.001).

**Results:**

The total mean score of the participants was 10.6±1.5, ranging from 7 to 13. 58(93.5%) students had poor triage knowledge. In the scenario’s section, the percentage of correct triage by students was 49.2% and those of over and under triage were 28.1% and 22.7%, respectively. There was a significant relationship between the triage accuracy and level of triage (ESI 4) (p≤0.001).

**Conclusion:**

The level of knowledge of triage in the last year medical students was poor, although most of them had passed a course in the screen room. It is recommended that medical students’ educational courses should include sections on the knowledge of triage in emergency rooms.

## Introduction

Timeliness of clinical care is essential for reduction of mortality and morbidity in medical centers. Seconds and minutes can determine the difference between death and disability and a healthy life in Emergency Departments (EDs) (1). The main expectation from the EDs is providing immediate medical care appropriate to the severity of clinical status of the patients. Overcrowding threatens the delivery of safe and timely care to patients (2). EDs are not well developed in Iran and overcrowding is a major problem in the country. Increasing the number of Emergency Medicine (EM) trained physicians practicing in the EDs is promoted by authorities with the goal of increasing the country’s ED capacity and capabilities, so a 4 week course has been implemented in the internship program of the last year medical students since 2005 (3).

Triage is a response to the problem of overcrowding in EDs and is a decision making process to accelerate rapid identification of critically ill patients from those with non-urgent complaints and prioritize the patients based on the severity of clinical status (4). Employment of triage in EDs has considerably improved the waiting times for emergency care and increased patients' satisfaction with EDs (5). Precision, accuracy and timeliness of decisions made by the triage unit affects the ultimate outcome of EDs (6).

Knowledge of Triage has been investigated previously among various health care professionals (7-10). Hedayati et al. (2011) in their study concluded medical students’ low level of knowledge about hospital triage can be due to lack of exclusive triage education course and not having emergency departments which follow evidence-based decision making (11).

Tabatabai et al. (2015) in their study concluded the low awareness scores of the studied students about triage highlighted the need for more supervision during internship, emphasis on triage in university courses, and specialized triage training courses for the students (12).

Last year medical students (7^th^ year) in Shiraz University of Medical Sciences attend a course in the screen room in EDs of Nemazee and Shahid Faghihi hospitals. Because some of these students are employed in EDs after graduation and their knowledge of triage can directly affect their performance in EDs, we conducted this study to examine their knowledge of triage and determine their performance in paper-based triage case scenarios.

## Methods

This prospective survey was conducted in Nemazee and Shahid Faghihi hospitals of Shiraz, Iran. From January to June 2013, 62 last year medical students of Shiraz University of Medical Sciences who attended emergency medicine course in the screen room of the two hospitals were examined. In both hospitals, the last version of 5 level triage scale Emergency severity index version 4 (ESI 4), adopted by the National Center for Disaster Management and Medical Emergencies to be implemented in hospital EDs nationwide is used with some modifications. In this triage system, the patients are categorized into 5 priorities based on the severity of the illness, degree of urgency and resource requirement:  ESI-1 being the most unstable, urgent and resource intensive, and ESI-5 being the least (4).

After obtaining the approval of Medical Ethics Committee in Shiraz University of Medical Sciences t, medical students were asked to voluntarily participate in the study. We used a questionnaire designed by national committee on triage education and promotion; it has been previously used in national studies frequently and its validity and reliability have been confirmed (6). The questionnaire has been designed in 3 sections: section one includes personal information regarding age, sex, average mode of introduction to triage, and previous experience of triage in ED. Section 2  (Triage Knowledge Section) consists of 15 correct/incorrect questions regarding the participants' knowledge of triage with 3 choices including: correct, incorrect, and don't know. Each correct answer receives the score 1, and other choices receive the score 0. The total scores were calculated for each participant and classified into 3 levels: poor (less than 50% correct answer), intermediate (50 to 75%) and strong (more than 75%). In section 3 (Scenarios section), ten case scenarios were introduced and the students were asked to assign a priority level to each case. The case scenarios included 1 level I patient, 2 level II and level III patients, 1 level IV and 3 level V patients. Two pediatric and one obstetric cases were included in scenarios. The rate of correct triage, over-triage (assigning a triage level greater than the expected level), and under-triage (assigning a triage level lower than the expected level) were calculated for each participant.

The questionnaires didn't include any instruction, and were completed without help or guidance from others. All questionnaires were anonymous and the students who were not willing to participate were excluded from the study. Statistical analysis was performed using SPSS statistical software (version 14). Comparisons of the calculated scores were made using independent sample t-test, one way ANOVA. p≤0.001 was considered as statistically significant. 

## Results

The questionnaires were filled out by 62 medical students, 51.5% were females. The students’ mean age was 23.9±0.9 years. 80% of the students had passed their emergency medicine course and had encounters with triage. 

The mean total score of the participants in triage knowledge was 10.6±1.5, ranging from 7 to 13. Triage knowledge scores were categorized as intermediate in 8 students (12.9%) and poor in 54 students (87.1%). Mean knowledge score based on 15 questions of Triage Knowledge Section (section 2 of questionnaire) was 5.7 1.2, and 58 students (93.5%) were categorized as poor and 4 students (6.5%) as intermediate knowledge. The total scores of triage knowledge section did not show a statistically significant relationship to gender (p=0.015), grade point average (p=0.078), and history of triage experience (p=0.061).

In section 3 (Scenarios section), the percentage of correct answers (correct triage) was 48.2%. 38(61.3%) students answered correctly to more than 50 percent of the questions, and only 1(1.6%) student had more than 75% correct answers. Among incorrect answers of this section, over-triage and under-triage answers were 28.1%, 22.7%, respectively.

Performance of students in different levels of triage is summarized in Table 1. In 2 scenarios, all the students had correctly categorized the case (one level I question –cardiac arrest- and one level V case –re-prescribing antidepressant agent). The highest rate of accurate triage after these two cases was for one level V case –ommon cold (53%). In one level V case –minor head trauma- all students had wrong answer (54.8% had chosen level III and 45.2% had chosen level IV), and the highest rate of wrong answers after this case was for one level III case –abdominal pain in a dialysis patient (72.6%). There was a significant relationship between triage accuracy and level of triage (ESI 4) (p≤0.001).

**Table 1:** Rate of correct triage, over-triage and under-triage in different levels

**Level of triage**	Level I	Level II	Level III	Level IV	Level V	All levels
**No of questions**	1	2	2	2	3	10
**Total no of answers**	62	124	124	124	186	620
**Correct Triage n(%)**	62(100)	52(41.9)	38(30.6)	60(48.4)	93(50)	305(49.2)
**Under triage n(%)**	0	44(35.5)	61(49.1)	36(29)	0	141(22.7)
**Over triage n(%)**	0	28(22.6)	25(20.2)	28(22.6)	9 (50)	174(28.1)

## Discussion:

Our findings indicate that the knowledge of triage among last year medical students in Shiraz University of Medical Sciences is poor, although most of these students had previous experience with triage in ED and screen room, only 49.2% of the triages performed by students were correct.

Performance of students in triage of level I and V patients (100 and 50%, respectively) was better than middle levels. 100% correct triage in the level I case demonstrates the ability of students in identifying critically ill patients, although high percentage of correct triage in level I and V patients may be due to the easier recognition of patients in the two extremes of the spectrum of severity of the illness. 

In a study on emergency pre-hospital health care professionals, triage scores were 13.03 out of 20 (equal to 65.15%) for physicians and nurses and 11.83% (equal to 59.15%) for emergency paramedics. All professional groups tended to over-triage patients, and there was no significant difference in the scores received by both doctors and nurses, but paramedics did not do as well as both nurses and doctors (8). In another study by Considine et al., 61% of the triage decisions made by the nurses were correct, 18% were under-triage decisions, and 21% were over-triage decisions. In this study, the highest and lowest percentages of correct triage were for levels III and II, respectively (9). Triage knowledge of medical students has been proven to be poor (less than 50%) in previous studies (10). Triage accuracy of the first year medical students after receiving a brief training session was 64.3%; the overall rate of over-triage was 17.8%, compared to an under-triage rate of 12.6%. These students obtained triage accuracy scores similar to those of emergency physician and registered nurses (11).

The rate of over-triage and under-triage in the present study was 22.7% and 28.1%, respectively. The highest rates of under-triage were in the levels II and III, (35.5% and 49.1%, respectively) and the highest rate of over-triage was in the level V (50%). Wrong answers in a study by Mirhaghi and colleagues included 48% over-triage and 57.8% under-triage. In this study, a high rate of under-triage which directly threatens the patients’ safety and health has been reported in participants with higher scores (23% for high score participants vs. 15% for low score participants) (13). In our study, the high rate of under-triage in level III can be harmful by ignoring the high risk patients. Although a high rate of over-triage does not directly cause harm to patients, but it leads to overcrowding in EDs and prevents timely care of acutely ill patients.

In our study, most of the students had passed the emergency medicine course and attended screen section of ED and had directly attended the triage setting, so they were expected to have a better performance in triage. The most significant reason for poor performance of these students seems to be lack of appropriate education on hospital triage. In a study assessing the accuracy of triage among army nurses, the most significant factors associated with higher scores on the Triage Test were completion of ACLS and related courses for emergency and critical care nursing (13).  In another study teaching methods based on pattern recognition skill development optimized triage performance of healthcare students (14). Our questionnaire didn't have any instruction on triage, while the use of triage decision-making materials in questionnaires has resulted in a significant increase in correct responses of police firearm officers in pre-hospital triage, and this improvement in accuracy resulted mainly from a reduction in the extent of under-triage (15).

Considine and colleagues reviewed the independent roles of factual knowledge and experience in triage decisions in numerous studies and concluded that factual knowledge is an important factor in improving triage decisions and although a number of studies have examined the role of experience as an independent influence on triage decisions, none has found a significant relationship between experience and triage decision-making, and factual knowledge appears to be more important than years of emergency nursing or triage experience in the accuracy of triage decisions (16-17).

## Conclusion

Medical education for medical students in Shiraz University of Medical Sciences is obviously lacking triage training. Incorporating a chapter for teaching triage to emergency medicine course of medical students and practical training in ED is recommended to enhance the knowledge and experience of this pivotal group, using role modeling in Emergency Department in performance of EDs with the aim of improving the overall performance and functioning of EDs and hospital triage.  

**Limitations**

Medical students voluntarily participated in the study; although most students agreed to complete the questionnaires, this caused a sample selection bias which limits the genaralizabilty of our findings. Also,the paper based nature of written case scenarios does not provide an ideal simulation of real cases and students may make better decisions in real situations. Providing cases with multimedia objects in computer-based scenarios is recommended for future studies.

## Notes

**Source of funding:**none declared.
